# Risk and Cooperation: Managing Hazardous Fuel in Mixed Ownership Landscapes

**DOI:** 10.1007/s00267-012-9848-z

**Published:** 2012-04-11

**Authors:** A. Paige Fischer, Susan Charnley

**Affiliations:** 1 US Forest Service, Western Wildland Environmental Threat Assessment Center, Pacific Northwest Research Station, 3200 SW Jefferson Way, Corvallis, OR 97331 USA; 2 US Forest Service, Pacific Northwest Research Station, Portland, OR USA

**Keywords:** Wildfire risk perception, Cooperation, Landscape management, Nonindustrial private forest owners, Multi-method design, Logistic regression, Qualitative analysis, Social exchange

## Abstract

Managing natural processes at the landscape scale to promote forest health is important, especially in the case of wildfire, where the ability of a landowner to protect his or her individual parcel is constrained by conditions on neighboring ownerships. However, management at a landscape scale is also challenging because it requires cooperation on plans and actions that cross ownership boundaries. Cooperation depends on people’s beliefs and norms about reciprocity and perceptions of the risks and benefits of interacting with others. Using logistic regression tests on mail survey data and qualitative analysis of interviews with landowners, we examined the relationship between perceived wildfire risk and cooperation in the management of hazardous fuel by nonindustrial private forest (NIPF) owners in fire-prone landscapes of eastern Oregon. We found that NIPF owners who perceived a risk of wildfire to their properties, and perceived that conditions on nearby public forestlands contributed to this risk, were more likely to have cooperated with public agencies in the past to reduce fire risk than owners who did not perceive a risk of wildfire to their properties. Wildfire risk perception was not associated with past cooperation among NIPF owners. The greater social barriers to private–private cooperation than to private–public cooperation, and perceptions of more hazardous conditions on public compared with private forestlands may explain this difference. Owners expressed a strong willingness to cooperate with others in future cross-boundary efforts to reduce fire risk, however. We explore barriers to cooperative forest management across ownerships, and identify models of cooperation that hold potential for future collective action to reduce wildfire risk.

## Introduction

Boundaries: fires don’t understand them. We can’t draw a line and say we did our part up to this point, and now we are good…It’s just a bigger picture.

This forest landowner from eastern Oregon recognizes that fire occurs on a landscape scale. Although he believes people need to manage fire risk beyond their property lines, he has not cooperated with any of his neighbors to address hazardous fuel conditions locally. “We communicated with them…but they have their own balance of what they want to do,” he explained, referring to gulfs in values and priorities for forest conditions and management. This landowner thins thickets of trees but leaves brush for deer forage. He is concerned that one of his neighbors eliminates too much habitat in his efforts to reduce fuel, while another does nothing.

The importance of managing natural processes and biodiversity at the landscape scale to promote the health and productivity of forest ecosystems is widely recognized (e.g., Lindenmayer and Franklin 2002). Doing so, however—especially when it entails managing across ownership boundaries—remains challenging. Different land ownerships, public and private, are managed for different goals using different actions, with differing ecological effects (Landres and others 1998). In the case of fire, hazardous fuel reduction on one ownership can reduce the risk of fire on neighboring lands. Similarly, suppression activities on one ownership can cause fire to be excluded from another ownership, causing fuel buildups that can lead to uncharacteristically severe fires having dire social, economic, and ecological consequences. Where management activities have ecological, economic, or social consequences beyond ownership boundaries, and the efficacy of one landowner’s actions can be limited or improved by those of nearby landowners, cooperation can be an important strategy for achieving landscape-scale management goals (Yaffee and Wondolleck 2000). Cooperation is also an alternative to regulation for the management of common pool resources such as forests; local residents who develop voluntary, self-regulating management institutions may have greater expertise and incentive for managing these resources effectively than regulatory agencies (Ostrom 1990). Yet the decision to cooperate with others hinges on a balance between altruism and self-interest, and in this case, on whether landowners are willing to accept the immediate burden of cooperating with others in exchange for the longer term, but less certain, benefit of buffering their properties against fire.

In this paper we explore the relationship between nonindustrial private forest (NIPF) owners’ perceptions of fire risk, including risk associated with conditions on nearby forestlands (landscape-scale risk), and their decisions to treat hazardous fuel in cooperation with others. Our study area is the ponderosa pine (*Pinus ponderosa*) ecotype on the east side of Oregon’s Cascade Mountains, where a history of fire suppression, grazing, and timber harvest has led to a buildup of hazardous fuel and thus, fire risk (Hessburg and others 2005). Although this area is dominated by federal lands, NIPF owners own 1/6th of the forestland in the area. Much of their land borders or is near federal land, creating a mixed-ownership landscape in which their management practices affect the connectivity of fuel, and potential movement of fire, between federal wildlands and populated areas (Ager and others 2012).

Given that fire does not observe ownership boundaries, and that fuel conditions on one ownership can affect fire risk on neighboring ownerships, we hypothesized that owners who perceive a risk of wildfire to their properties, and perceive that conditions on nearby forestlands contribute to this risk, are more likely to cooperate with others to reduce fire risk across ownership boundaries. We expected owners to be motivated by the rationale that cooperation would enable them to accomplish fuel reduction activities more efficiently together than alone. Yet we also expected that social beliefs and norms about cooperation and private property ownership would influence owners’ decisions to treat fuel through cooperation with others.

We investigated the relationship between risk perception and cooperation through statistical analysis of mail survey data. We used qualitative interview data to examine how NIPF owners perceive fire risk on their own properties and on the wider landscape, and communicate and cooperate with other private and public owners to address fire risk. Interview data also allowed us to explore the influence of individual beliefs, social norms, and institutions on cooperative fuel treatments, and to identify potential models of cooperation. After presenting our results, we discuss barriers to cross-boundary cooperation in hazardous fuel reduction and ways to potentially overcome them. The ecological and socioeconomic conditions prevalent in our study area are common throughout the arid West. Thus, this case from eastern Oregon may shed light on opportunities for managing fire-prone forests using an “all lands approach” elsewhere in the West.

## Literature Review

### Risk Perception

Risk perception, defined as the “subjective probability of experiencing a damaging environmental extreme” (Mileti 1994), is considered an important antecedent to mitigation and adaptation behavior according to the natural hazards literature (Paton 2003). In the case of wildfire and other natural hazards, risk perception has been identified as a key variable influencing mitigation behaviors such as taking action to reduce hazardous conditions, preparing for a hazardous event, or moving to a less hazardous area (Dessai and others 2004; Grothmann and Patt 2005; Amacher and others 2005; Niemeyer and others 2005; Jarrett and others 2009; McCaffrey 2004; Fischer 2011; Winter and Fried 2000).

People form perceptions of risk through interaction with friends, peers, professionals, and the media on the basis of norms, world views, and ideologies (Douglas and Wildavsky 1982; Berger and Luckmann 1967; Tierney 1999). The process of coming to agreement on the causes and consequences of risk, and acceptable levels of uncertainty and exposure, is influenced by the level of legitimacy and trust between people and institutions (Slovic 1999). Cognitive biases (e.g., discounting future events, giving disproportionate weight to vivid or rare events, and denying risk associated with uncontrollable events) also play a role in risk perception (Maddux and Rogers 1983; Slovic 1987; Sims and Baumann 1983), as can people’s past experience and objective knowledge (Hertwig and others 2004).

However, risk perception alone does not always compel mitigation behavior. Other important variables include believing one is capable of acting to effectively mitigate risk, holding oneself responsible for one’s welfare, and feeling sentimental attachment to a vulnerable community or place (Paton 2003). Moreover, decisions to mitigate risk occur under complex socioeconomic conditions that both shape people’s vulnerability to risk (Slovic 1999), and determine their efficacy at addressing risk (Slovic 1987; Maddux and Rogers 1983; Tierney 1999).

### Cooperation

Cooperation refers to a spectrum of behaviors that range from communicating with others about shared interests to engaging in activities that help others, including sharing resources and work (Yaffee 1998). The theory of cooperation is based on the benefits of reciprocity to participating parties when combined efforts can achieve more than individual efforts. Disciplines ranging from evolutionary biology to political science have examined cooperation as a response to adverse and unpredictable environments, and as a strategy for hedging against and coping with environmental risk (Andras and others 2003; Ostrom 1990; Cohen and others 2001; Axelrod and Hamilton 1981). Social conditions that foster cooperation among individuals include the presence of common goals and motivations, a perception of common problems (including risks), the use of similar communication styles, high levels of trust, and expectations and opportunities for frequent exchanges of information and ideas (Yaffee 1998; Bodin and others 2006; Ostrom 1990). Policy environments, land tenure arrangements, and power relations must also be conducive to cooperation (Ostrom 1990; Bergmann and Bliss 2004).

Three important antecedents to cooperation, including cross-boundary cooperation among private landowners, are shared cognition, shared identity and legitimacy (Rickenbach and Reed 2002; Gass and others 2009). Shared cognition refers to sharing a similar perspective or having consensus on a problem or task (Bouas and Komorita 1996; Swaab and others 2007). Shared identity means sharing membership in a community or social group (Tyler 2002; Tyler and Degoey 1995; Swaab and others 2007). Legitimacy is when people or organizations are viewed as fair and capable and are empowered by others (Tyler 2006).

Social exchange theory provides a framework for understanding when cross-boundary cooperation by NIPF owners might occur. Social exchanges are interdependent interactions among people that generate mutual benefits and obligations. One type, “reciprocal exchanges”, consists of interactions that lack terms or assurance of reciprocation (Blau 1964). Reciprocal exchanges are an informal form of cooperation that functions on the basis of reciprocity rules (an action by one party leads to an action by another party), beliefs (that people who are helpful now will receive help in the future), and norms of behavior (that people should reciprocate based on social expectations) (Molm 1994; Cropanzano and Mitchell 2005). Reciprocal exchanges entail risk and uncertainty because they occur in the absence of a contract. When they are successful, they yield trust and commitment, which in turn lead to stronger relationships (Blau 1964). When they are unsuccessful, cooperation breaks down. In contrast, “negotiated exchanges” are social exchanges that have known terms and binding agreements to provide some assurance against exploitation (Coleman 1990). Negotiated exchanges do not entail as much risk or require as much trust as reciprocal exchanges (Molm and others 2000).

The risks associated with cooperation increase when “mismatches” occur between the nature of the relationship among the cooperators and the nature of the transaction between them (Cropanzano and Mitchell 2005). For example, when two landowners who have an interpersonal relationship (one that depends on obligations, trust and interpersonal attachment) engage in an economic exchange (an exchange of goods or services), there is a mismatch. In such cases, people who act to the economic benefit of others may feel betrayed if that economic benefit is not reciprocated, and may be reluctant to enter into another such relationship. Thus, neighboring landowners who have an interpersonal relationship and who cooperate in fire risk reduction activities—which are economic because they entail investment of one person’s resources in the protection of another’s property—have a mismatch, exacerbating the risks associated with cooperation. We return to these observations in our Discussion.

## Methods

### Definitions

Our construct of wildfire risk perception among NIPF owners includes concern about a wildfire occurring on one’s land, and concern about hazardous fuel conditions on nearby private or public land contributing to the chance of wildfire on one’s land, based on Mileti’s (1994) definition of risk perception as subjective probability. We also included awareness of the ecological role of wildfire in ponderosa pine forests, and past experiences with wildfire on one’s property as elements of our risk perception construct based on Hertwig and others (2004). For purposes of our analysis, we define cooperation as jointly planning, paying for, or conducting activities that reduce hazardous fuel. We focus on cooperation among NIPF owners, and between NIPF owners and public agencies.

### Data Collection

In September 2008 Oregon State University and Oregon Department of Forestry funded and administered a mail survey to owners of a random sample of NIPF parcels in eastern Oregon’s ponderosa pine ecosystem. The goal of the survey was to learn more about NIPF owners’ wildfire management practices, constraints on fire management, and how public agencies could design better assistance programs.

**Fig. 1:**
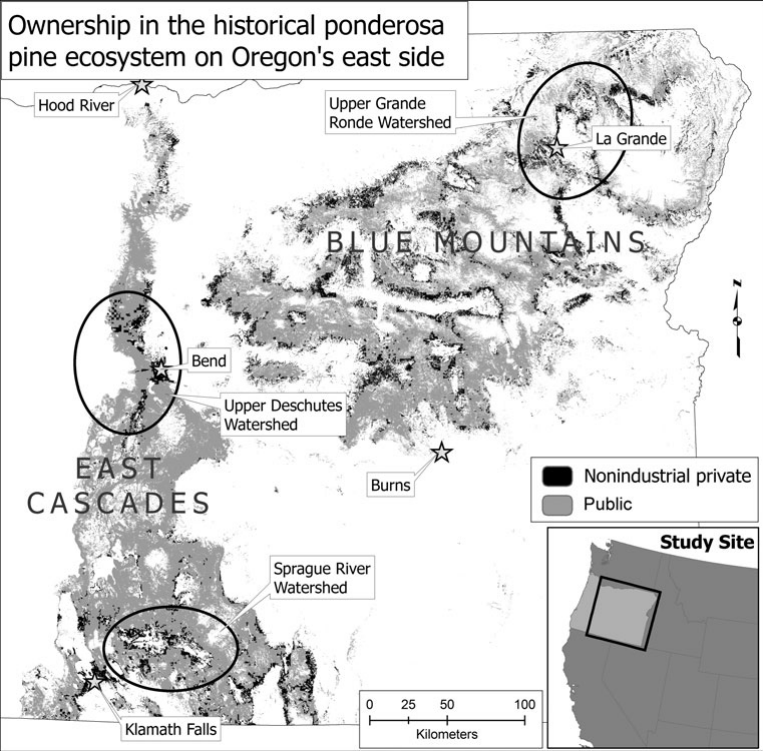
Study area showing nonindustrial private forest and public ownership and case–study locations

The survey sample was selected by casting random points across a GIS polygon created using layers of pixels that represent historical and potential ponderosa pine forests (Grossmann and others 2008; Ohmann and Gregory 2002; Youngblood and others 2004) and an ownership layer (Fig. 1). The NIPF polygon comprised approximately 1.2 million hectares, about 50 % of all NIPF land and 15 % of all forestland east of the Cascade Range in Oregon, which is consistent with other estimates of the proportion of land in NIPF ownership in eastern Oregon (Oregon Department of Forestry 2006). The point layer was joined with a state tax lot layer obtained from the Oregon Department of Revenue to create a list of owner names, addresses and tax lot numbers.

The survey asked about owners’ past (2003–2008) and intended future (2008–2013) hazardous fuel reduction activities, including cooperation with public agencies, nonprofit organizations, private consultants or other private landowners. Survey questions also addressed owners’ goals, experiences with wildland fire, concern about fire risk in general, concern about specific hazards and potential losses, and demographic characteristics. Respondents were asked to reference the parcel associated with the tax lot number on their survey. The survey was reviewed by 20 natural resource professionals, landowners, and social scientists and approved by the Oregon State University Institutional Review Board prior to implementation.

The survey was administered to 1,244 owners using the total design method (Dillman 1978): an announcement card, followed five days later by the survey; a second survey to non-respondents 2 weeks after the first; and at week four, a thank you card that also served as a final reminder to non-respondents. Of the 1,244 surveys mailed, 234 were disqualified, leaving 1,010 valid surveys. From these, we received 505 valid responses, yielding a response rate of 50 %. No follow-up survey of non-respondents was conducted.

The survey respondents consisted mostly of retirement-age males, similar to NIPF owners in the American West (Butler and Leatherberry 2004), but more had obtained bachelor’s degrees, earned above the national median household income ($50 K), and were absentee (Butler and Leatherberry 2004). Also, a high proportion had treated their parcel to reduce the risk of wildfire compared to owners in the West generally (Brett Butler, unpublished National Woodland Owner Survey data 2006). They also owned relatively large holdings compared to other owners in the West (Butler and Leatherberry 2004). These disparities reflect the sampling approach (based on forestland, not forest owners), and the social and biophysical conditions in eastern Oregon where land use rules set large minimum tax lot sizes, and arid climate limits productivity, favoring forestry and grazing over large areas. These and other characteristics of the sample are presented in Table 1.

**Table 1:** Characteristics of survey sample (*n* = 505)

Female (percentage)	20.4
Bachelor’s degree (percentage)	51.7
Earn at least U.S. median income of $50 K (percentage)	73.5
Age (mean)	63.1
Use parcel as primary residence (percentage)	25.5
Distance of parcel from primary residence in miles (median)	75.0
Most important management goal is “residence” (percentage)	20.0
Years parcel owned (mean)	21.7
Parcel acreage (median)	392.0
Ownership acreage (median)	540.0
Treated acres to reduce risk of fire (percentage)	70.0
Acres treated (median)	20.0

We conducted semi-structured key informant interviews in 2007 and 2008 with a purposive sample of 60 NIPF owners owning forestland in three watersheds in the study area that are considered high priority for hazardous fuel reduction (Oregon Department of Forestry 2006): the Sprague, Upper Deschutes, and Upper Grande Ronde (Fig. 1). We identified owners having diverse fire experiences, management intensities, and ownership characteristics with help from local natural resource agencies and organizations. Each interview included a walking tour of the owner’s property and averaged two hours. Questions addressed their management approaches, experiences and concerns with fire, ecological knowledge and values about fire and forest conditions, and perceptions of opportunities and constraints for hazardous fuel reduction. Most interview informants had treated some portion of their parcel to reduce the risk of wildfire. Digital recordings of the interviews were transcribed verbatim and entered into Atlas.ti, a software program that aids qualitative data analysis. The interview sample was similar to the survey sample in terms of demographic characteristics.

### Data Analysis

To analyze the mail survey data we used frequencies to describe respondents’ perceptions of fire risk and their cooperation behaviors, and logistic regression to identify the relationship between risk perception, and cooperation on fuel reduction. We began the logistic regression analysis with a manual backward stepwise regression of the cooperation variables on the risk perception variables and a set of demographic control variables, and then built final models with the variables that were relevant to the hypothesis. Table 2 contains descriptions of the cooperation response variables and risk perception explanatory variables.

**Table 2:** Variables used in logistic regression tests

Variable	Type	Definition
Cooperated with public agencies	Dichotomous response	Worked with public agencies to plan, pay for, or conduct practices that can reduce hazardous fuel on their parcels: 1 if yes; 0 otherwise
Cooperated with private owners	Dichotomous response	Worked with other private owners to plan, pay for, or conduct practices that can reduce hazardous fuel on their parcels: 1 if yes; 0 otherwise
Willing to cooperate with public agencies	Dichotomous response	Willing to work with public land neighbors to reduce fuel with the expectation that cooperation will fulfill at least one of the following conditions: (a) reduce treatment costs, (b) increase acreage treated, (c) make more equipment available, (d) make more funding available, (e) make more training and education available, or (f) provide an exemption from legal liability for escaped fires: 1 if yes; 0 otherwise
Willing to cooperate with private owners	Dichotomous response	Willing to work with private land neighbors to reduce fuel with the expectation that cooperation will fulfill at least one of the following conditions: (a) reduce treatment costs, (b) increase acreage treated, (c) make more equipment available, (d) make more funding available, (e) make more training and education available, or (f) provide an exemption from legal liability for escaped fires: 1 if yes; 0 otherwise
Concerned about fire occurring on parcel	Dichotomous explanatory	Five-point scale of concern about wildfire occurring on parcel: 1 if concerned or very concerned; 0 if not at all concerned, slightly concerned or moderately concerned
Concerned about hazard on nearby public land	Dichotomous explanatory	Five-point scale of concern about conditions on nearby public land contributing to the chance of wildfire on parcel: 1 if concerned or very concerned; 0 if not at all concerned, slightly concerned or moderately concerned
Concerned about hazard on nearby private land	Dichotomous explanatory	Five-point scale of concern about conditions on nearby private land contributing to the chance of wildfire on parcel: 1 if concerned or very concerned; 0 if not at all concerned, slightly concerned or moderately concerned
Aware of local fire ecology	Dichotomous explanatory	Agree with statement “wildfire can help maintain open, park-like conditions that are characteristic of ponderosa pine forests”: 1 if yes; 0 otherwise.
Experienced a fire on parcel	Dichotomous explanatory	Experienced a wildfire on parcel, or lost trees of value, or lost structures, or lost a home to a wildfire on parcel: 1 if yes; 0 otherwise

To analyze the interview transcripts we followed a standard protocol of qualitative analysis (Patton 2002). We identified and coded quotations in the transcripts that provided evidence for how interview informants perceive fire risk, including the probability of fire, the hazardous conditions that contributed to the probability of fire, and what values they were concerned about losing in the case of fire. We also coded quotations that provided evidence for how owners view the barriers and opportunities of cooperation. We linked these quotations with additional codes and wrote memos about how wildfire risk perceptions motivated owners to cooperate with others.

## Results

### Risk Perception and Hazardous Fuel Management

We are always concerned about fire. Our fear every summer is where is the lightning strike going to be and are we going to be able to survive the fire? That is one of the reasons we created fire breaks throughout the property, and because our neighbors didn’t have any.

**Fig. 2:**
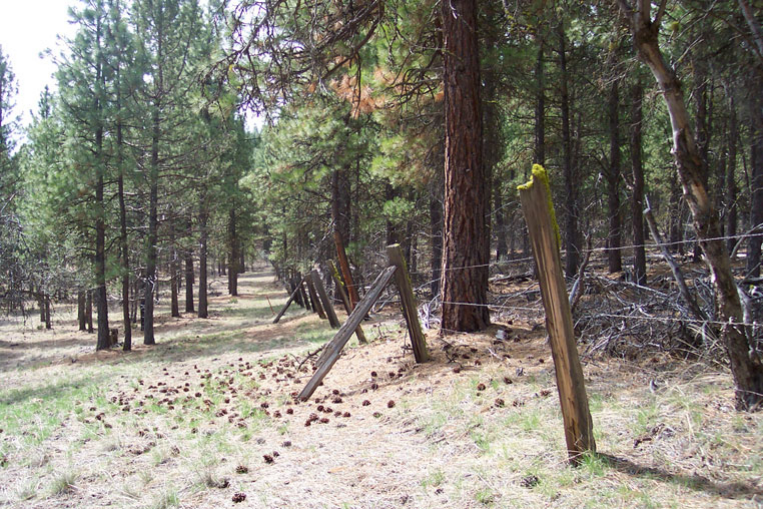
Property boundary: private on *left*, public on *right*

Comments like this one indicate that some landowners interviewed were aware of fire risk beyond their property boundaries, and responded by treating fuel. Survey responses corroborated this finding. 67 % of the survey respondents said they were concerned about a fire affecting their property. A majority (53 %) were concerned about conditions on nearby public lands contributing to the risk of wildfire on their property. Interview informants articulated similar concerns, although few were aware of which land management agency controlled nearby public lands. “You want to see risk? There’s risk,” responded one interviewee when asked for an example of hazardous forest conditions. Like many owners we interviewed, he pointed to land on the other side of his fence line, in this case national forest land in the Sprague River Watershed. “Here you can see where it is thinned and then it gets really thick; that is a piece of government ground. That is the difference between my place and the government ground; theirs is jungle.” Figure 2 shows forest conditions we often encountered across property lines owners shared with federal land management agencies.

Some owners were also concerned about fuel conditions on neighboring private lands, as evidenced in this comment by another interviewee from the Sprague River Watershed: “That is an inferno waiting to happen…He’s endangering my property, my structures, and also my forest”. However, owners were less concerned about conditions on nearby private lands than on nearby public lands. Only 37 % of survey respondents were concerned about fire risk from nearby private lands. Some interview informants believed that most private owners managed their forests enough (i.e., thinned and harvested) that little fuel was left to be of consequence. “They are logging the living daylights out of that,” exclaimed one interviewee, referring to the surrounding industrial ownership. “It’s going to be fine for a lot of years.” Other interviewees were simply more forgiving about the risk associated with private lands than with public lands. One owner guessed that her neighbors “are doing fine…doing it about the same way we are: thinning, logging it every few years…The cattle are keeping the brush down.”

70 % of the survey respondents had treated portions of their parcels to reduce the risk of fire between 2003 and 2008. They used a range of forest management practices that can reduce fuel, presented in Table 3. The median treatment area was 20 acres (interquartile range = 1–120 acres). Many interviewees said that they treated their properties to compensate for the lack of hazardous fuel management by their neighbors. As one owner in the Sprague River Watershed explained,

**Table 3:** Management practices of sample (*n* = 505)

Management practice	Percentage of respondents who conducted practice on their parcel between 2003 and 2008
Burned material in piles	65.5
Grazed livestock	65.5
Thinned by hand or chainsaw	64.6
Pruned or limbed up trees	60.9
Cleared around structures	50.2
Created fuel breaks	48.1
Made structures more fire proof	42.1
Pulled plants, brush or trees by hand	41.0
Mulched, spread or left material in the forest	38.3
Thinned with mechanized equipment	36.4
Mowed, crushed, ground or chipped	33.5
Applied herbicides	32.0
Harvested timber for profit	28.7
Understory burned	21.8
Sold logs for profit	19.9
Sold wood products for profit	12.1
Planted fire-adapted trees	11.1
Took material to landfill	7.5

If we have a higher risk because of heavy fuel build-up on adjacent land…we look at our management philosophy a little bit differently. We would do more in our cutting, more than we like…to keep a crown fire from spreading.

Indeed, in a different analysis of the survey findings we found that owners’ concern about fire risk, and concern about conditions on nearby public land contributing to this risk explained their likelihood of treating fuel (Fischer 2011).

### Risk Perception and Cooperation

Most owners worked either on their own or with family members, or with private contractors to conduct forest management activities. However, many had also worked in cooperation with others. Between 2003 and 2008, 34 % of the survey respondents cooperated with public agencies, 18 % cooperated with other private owners, and 15 % cooperated with nonprofit organizations to plan, pay for, and/or conduct practices that can reduce fuel (Table 4).

**Table 4:** Past cooperation in forest management activities (*n* = 505)

Arrangement	Percentage of respondents who used arrangement
Only on one’s own or with family members	35.0
With public agencies, other private owners or nonprofit groups	41.2
With public agencies (e.g. ODF, BLM, NRCS)…	33.8
With private forest owners (e.g. neighbors)…	17.8
With nonprofit groups (e.g. watershed councils)…	14.8
With private contractors	41.0

Interview informants provided examples of cooperative fuel treatment, particularly with public land neighbors: participating in fire management planning with the Forest Service and the Bureau of Land Management for lands adjacent to their properties; communicating with agencies about the need to reduce fuel along shared property boundaries; coordinating forest thinning and brush-clearing with treatments on adjacent public lands to widen fuel breaks; and synchronizing prescribed burns with those on adjacent public lands to take advantage of agency fire fighters and equipment.

Interview informants cited fewer examples of cooperation with private landowners. These included allowing neighbors to graze livestock on their properties to reduce grass and brush, and planning treatments along shared property boundaries to create wider, shared fuel breaks. More often they observed the use of new techniques or equipment on each other’s parcels. A number of owners said they had referred interested neighbors to their consulting foresters or operators to request treatments similar to the ones performed on their properties. Thus, some portion of the 41 % of survey respondents who had worked with private contractors may have been influenced by, or influenced, other private owners, an indirect form of cooperation.

Owners expressed a greater willingness to cooperate with other landowners in the future to reduce fire risk than they had in the past. Most survey respondents said they would cooperate with both public owners (68 %) and private owners (75 %) to reduce fuel in the future, especially if it would release them from liability for fires resulting from escaped controlled burns, reduce their share of the cost of treatments, or make more public funding available to them for treatments (Table 5).

**Table 5:** Willingness to cooperate with other owners in the future (public or private) to reduce fire risk (*n* = 505)

Conditions under which respondents are willing to cooperate with other owners	Percentage of respondents willing to cooperate with other public or private owners under condition	
	Public owners	Private owners
Cooperation reduces liability	61.0	65.8
Cooperation reduces cost	53.9	58.6
Cooperation makes more public funding available	53.1	56.0
Cooperation makes equipment available	49.2	50.6
Cooperation increases acreage	49.2	49.6
Cooperation makes more training and education available	39.0	38.8
At least one of the above	67.7	74.7

According to the logistic regression tests, perceived risk explained cooperation between NIPF owners and public agencies, but not cooperation between NIPF owners and other private owners. Concern about a fire occurring on one’s parcel, and concern about conditions on nearby public land contributing to this risk were both associated (*P* ≤ .08) with whether owners reported having cooperated with public agencies in the past on forest management actions that can reduce fuel. Whether owners were aware of the historical role of fire in ponderosa pine ecosystems, and whether owners had experienced a fire on their land were also associated (*P* ≤ .05) with whether owners reported cooperating with public agencies in the past to reduce fire risk. Owners’ willingness to cooperate with public agencies in the future to reduce fire risk was also explained by the risk perception variables; specifically, whether owners were concerned about a fire occurring on their parcel (*P* ≤ .05), were concerned about conditions on nearby public lands and private lands (both at *P* ≤ .05), and were aware of the local fire ecology (*P* ≤ .05). None of the risk perception variables were associated with whether owners had cooperated with other private owners in the past. Only awareness of the local fire ecology was associated with their willingness to cooperate with other private owners in the future (*P* ≤ .01). *P* values and odds ratios for the risk perception variables are presented in Table 6. In addition, two demographic control variables were significant in preliminary manual backwards stepwise regression tests: living on one’s parcel and age were associated (*P* ≤ .05) with whether owners had cooperated in the past and were willing to cooperate in the future with both public agencies and other private owners, whereas parcel size, ownership size, tenure length, income, education and gender were not.

**Table 6:** Logistic regression predicting influences on cooperation (frequencies in parentheses)

	Dependent variables							
	Cooperated with public agencies (33.9)		Cooperated with private owners (17.8)		Willing to cooperate with public agencies (67.7)		Willing to cooperate with private owners (74.7)	
Independent variables	*P*	Exp(B)	*P*	Exp(B)	*P*	Exp(B)	*P*	Exp(B)
Concerned about fire occurring on parcel (67.3)	.012	1.941	.815	.935	.048	1.638	.218	1.387
Concerned about hazard on nearby public land (53.5)	.068	1.559	.311	1.335	.000	2.810	.214	1.396
Concerned about hazard on nearby private land (37.4)	.558	.867	.795	.928	.026	.551	.203	1.447
Aware of local fire ecology (65.5)	.049	1.621	.240	1.402	.005	1.959	.010	1.903
Experienced a fire on parcel (39.0)	.001	1.987	.659	.893	.430	.834	.890	1.035
Constant	.000	.130	.000	.175	.662	.893	.282	1.336
	Model χ^2^ = 31.194, Nagelkerke *R* ^2^ = 0.099		Model χ^2^ = 5.728, Nagelkerke *R* ^2^ = 0.021		Model χ^2^ = 29.973, Nagelkerke *R* ^2^ = 0.098		Model χ^2^ = 17.278, Nagelkerke *R* ^2^ = 0.062	

Our logistic regression test partially confirmed our hypothesis (owners who perceive a risk of wildfire to their properties, and perceive that conditions on nearby forestlands contribute to this risk, are more likely to cooperate with others to reduce fire risk across ownership boundaries). All of the variables included in our risk perception construct predicted past cooperation between NIPF owners surveyed and public agencies, and most predicted future willingness to cooperate with public agencies. In contrast, none of the risk perception variables predicted past cooperation between NIPF owners surveyed and other NIPF owners, and only awareness of the role of fire in ecosystems was associated with future willingness to cooperate among them. These findings indicate that other important influences on cooperation among private forest owners are at work.

### Barriers to Cooperation

Although many of the owners interviewed acknowledged the potential benefits of cooperation in fuel reduction—particularly for achieving economies of scale in their efforts—they identified numerous reasons for not cooperating. Barriers related to patterns of rural social organization were most commonly cited. “People in the timber sector are in an isolated spot,” explained an owner of 2,500 acres in the Sprague River Watershed, referring to the sparsely populated and mountainous landscape of Oregon’s east side, which impedes interaction. “[They] don’t have many neighbors [to cooperate with].” Furthermore, the markets and other natural resource-based economic activities that once provided a basis for interaction and reciprocity despite this topography are now in decline. An owner of 10 acres who recently moved to Union County in the Upper Grande Ronde Watershed explained:

When this place was small family ownerships primarily there was more talk between people and more helping each other out because they were all managing the land. Now people aren’t really deriving a significant amount of their income off the land…So they don’t tend to talk to each other or help each other out much.

As a result of demographic change, many newcomers own forestland primarily for privacy and solitude (Kendra and Hull 2005) or recreation. The isolation such owners seek counters interaction. “We’re like two separate little icebergs…we may touch…but only by necessity…it’s why we live out here,” explained an owner of 200 acres in the Deschutes River Watershed. A high rate of absentee ownership (74 % in our survey sample), often associated with recreational use, is a barrier to developing the social relationships upon which cooperation is predicated. Our regression results indicated that owners who live on their parcels were more likely to have cooperated with their neighbors in forest management than those who did not.

In addition, gulfs in values, beliefs, and motivations regarding the management of fire risk, also attributable to demographic change, were seen as barriers to cooperation. Owners who manage for commodities or habitat tended to view fire as a historically important and persistent ecological force. They believed hazardous fuel needed to be managed to prevent fire from being overly destructive, but did not seek to eliminate fire from the ecosystem. In contrast, owners who hold land primarily for residential reasons tended to view fire as a threat to their homes and scenic views, defining hazardous fuel as anything in the forest that could carry fire. Differing perceptions of fire and fuel led to conflicting approaches to forest management. For example, the owners of a 200-acre parcel in the Deschutes River Watershed selectively treated the most hazardous fuels in order to preserve wildlife and scenic beauty, differentiating themselves from their neighbors who razed all vegetation (apart from large overstory trees) within a 150-yard radius of their future home.

We understood their fire concerns, but we were also very concerned about how much they cleared out of the winter forage for the deer…We don’t want to see our forests be safe for wildfire but good for nothing else.

Conflict was especially apparent around fire treatments (conducting controlled burns, burning slash piles, and allowing naturally ignited fires to burn on one’s property). Some interviewees viewed fire as a tool for reducing risk associated with brushy, overstocked stands; others viewed fire as the risk itself. An owner of 10 acres in the Sprague River Watershed who managed primarily for habitat had permission to clear and burn brush on the property of his absentee neighbor. However, another neighbor with less risk tolerance stymied his efforts. “We had good conditions for burning,” he explained. “There were still snow drifts! Then these neighbors noticed what I was doing, got on the phone and threatened legal action. One guy threatened to kill me because they were so scared…And if you drive back there now you will see how much fuel there is; it’s scary.”

Conflicting values and goals relating to fire risk also impeded cooperation between NIPF owners and public land management agencies. An owner of 2,500 acres in the Sprague River Watershed was disappointed about a prescribed burn he had jointly conducted with the Forest Service, and attributed the problem to differing scales of risk tolerance. He believed the Forest Service was comfortable losing more trees in the burn than he was:

They were comfortable with a hotter controlled burn…than I was used to…For them this kind of mortality is nothing. They are dealing with thousands of thousands of acres. But when you [have] a limited number of acres, mortality has a different meaning.

Social norms about private property ownership and appropriate behavior towards neighbors were also identified by owners as constraints to cooperation, despite concerns about hazardous fuel conditions on neighbors’ lands. “I kind of try to hint to them,” said one interview informant, when asked why he hadn’t encouraged his next door neighbor to address hazardous fuel on his property. “But that is about as far as you can go because people are set in their ways.” The owner of 1,000 acres in the Upper Grande Ronde River Watershed was more direct: “If you want to have good neighbors you don’t mention things like that.”

Social norms about reciprocity, including the age-old challenge to collective action, free-ridership, also worked against cooperation. “The trouble with our society,” explained an owner in his 80s who controls hazardous fuel on his property despite being handicapped “is that one person can do the work…and other people will take the benefit.” In other words, if your neighbors reduce fuel on their properties, the risk to your property will be reduced without you having to do anything.

Owners were also concerned about potential risks to their autonomy as private property owners associated with participating in formal cooperative groups. For example, an owner of 650 acres in Klamath County recounted,

I have seen people—good friends—who aren’t speaking to each other today because they are in a big old group…It’s no longer: ‘Hey, Joe, come on over and help me fix my irrigation and I will come help you fix yours.’ It’s: ‘No I can’t come over because you have an inch more water than I do, and I don’t want to sue you about it.’—I don’t want to get into no organization.

Owners were also worried about participating in formal groups that include public agencies because of bureaucratic or regulatory burdens that might be imposed on them, and the discomfort of unequal power relationships. An owner of 200 acres in the Deschutes River Watershed, who had experienced frustration cooperating with federal agencies on fuel reduction and fish passage activities, explained: “it doesn’t feel good when you are feeling the heavy hand of government coming in saying you shall do this!” Nevertheless, about half of survey respondents declared membership in formal, natural resource-related groups (Table 7).

**Table 7:** NIPF organizational membership (*n* = 505)

Types of organizations	Percentage of respondents who said they belonged
Forestry organizations (OSWA, Society of American Foresters, etc…)	14.4
Fire fighting organizations (e.g. Forest Protective Associations)	18.4
Outdoor organizations (hunting clubs, fishing clubs, etc…)	24.5
Environmental organizations (Sierra Club, The Nature Conservancy, etc…)	18.8
Property or landowner’s association	16.2
Other similar organizations	4.6
An organization in at least one of the above categories	52.1

Finally, some owners mentioned laws that counter cooperation. The risk of being legally liable for fires or injuries resulting from negligent conditions or activities on one’s property discourages many owners from cooperating on fuel reduction work. “The problem is the law and the way liability is written,” explained one owner. “Nobody wants to be responsible.”

### Opportunities for Cooperation

We asked interviewees to describe cooperative arrangements for fuel reduction that would be amenable to them, based on their observations or experiences, and grouped their responses into three informal and three formal models that we then named.

In the informal, “over the fence” model, interviewees described landowners observing each other’s activities and doing something similar, or encouraging other landowners (often public agencies) to do more. Interviewees also suggested that owners could also jointly identify an issue that affects them and address it together (e.g., creating a fuel break). In the informal “wheel and spoke” model, contractors and other natural resource professionals help multiple nearby landowners learn indirectly from each others’ experiences, leverage financial resources, and access markets and fuel reduction services, without negotiating terms of cooperation among the landowners involved. In the “local group” model, interviewees described local change agents creating a forum in which landowners come together to address a common problem (e.g., the accumulation of hazardous fuel on nearby public lands). This informal process can lead to communication, cooperation, learning, and eventual leadership among members of the group. A number of interviewees claimed that informal models of cooperation are more effective than formal models because they don’t impose terms or require reciprocation, which can create adversarial relationships by establishing expectations.

Other landowners interviewed believed formal models of cooperation were more efficient and productive than informal models. In the “agency-led” model, interviewees described local natural resource management agencies providing education, technical, or financial support to help landowners learn from each other and interact around management activities; or, public funds so that landowners can implement fuel reduction themselves. In the “collaborative group” model, participants commit to a process and a product, are organized by a coordinator, and are guided by policy documents. Few owners had experience with formal “landowner cooperatives”. However, some proposed this model whereby groups of landowners would pool harvests and develop contracts with processers, working through a common contractor to increase their leverage in marketing biomass and small-diameter logs.

## Discussion

Cooperation is predicated on the benefits of reciprocity. People’s perceptions of risk can determine how they weigh the benefits and costs of working with others. This study finds that the majority of NIPF owners in Oregon east of the Cascade Mountains are concerned about fire risk to their properties, and beyond their property boundaries at a broad scale. Those who have cooperated with others in forest management activities that can reduce hazardous fuel are in the minority, however. Concern over fire risk did not appear sufficient to warrant cooperation with other private landowners in particular. Of course, some owners may lack concern about forest conditions on other private properties; a smaller proportion of owners were concerned about hazardous fuel conditions on nearby private lands than on public lands. And, some owners felt protected by heavy management on nearby private ownerships, especially industrial holdings. Nevertheless, roughly one-third of owners were concerned about the fire risk associated with other private ownerships, and the majority were willing to cooperate with other private owners in the future to mitigate that risk. That they have not acted on their concern in the past by trying to influence fuel conditions around them through coordinated planning and treatments with neighbors highlights the importance of other forces that work against cooperation. Here we draw on the literature presented earlier in this paper to discuss possible reasons for the disjuncture between NIPF owners’ ideals and behaviors regarding cooperation.

### Shared Cognition

Shared cognition is an antecedent to cooperation because it reduces the risk of participation. When parties to a collective effort perceive consensus among group members about the nature of the problem being addressed, the goals of the effort, and their commitment to the group, they are less likely to defect (Bouas and Komorita 1996; Swaab and others 2007). Although most NIPF owners surveyed perceived fire risk, it was clear in interviews that they did not hold common perceptions of wildfire, risk, or hazardous fuel. This lack of perceived consensus around the constructs of risk and hazard may hinder joint planning and implementation of fuel reduction activities. Some owners attributed their reluctance to cooperate to conflicting values and goals regarding forest conditions and perceptions of fire hazard and risk. However, awareness of fire as an important local ecological process was a predictor of willingness to cooperate with other private and public forest owners, suggesting that owners who share this view are more likely to cooperate.

Social exchange theory suggests that without shared beliefs about the probability and nature of fire risk, hazard, and the risk-reducing benefits of cooperation, owners may face difficulty rationalizing efforts to engage in potentially burdensome social relationships (Cropanzano and Mitchell 2005). This observation echoes what scholars of cooperation in the context of natural resources have argued: without a vision of a common problem or a common future, there is little reason to work together (Ostrom 1990; Yaffee 1998). Other studies of private forest owners have reached similar conclusions about the relationship between congruency of perceptions, attitudes and values, and joint planning (Rickenbach and Reed 2002; Jacobson and others 2000; Gass and others 2009).

### Group Membership

The constraints to cooperation that NIPF owners described in interviews were predominantly related to social organization: spatial isolation, a dearth of integrating economic activities, and social norms that inhibit communication and reciprocity among neighbors about fuel reduction. Survey findings that three-quarters of owners do not live on their properties provide additional evidence that social organization is a constraint on cooperation. Rural sociologists documented early on how topographical relief and spatial isolation influence social organization, and how resulting social relations affect the development of sociability (Field and Luloff 2002). Rural residents in eastern Oregon are spread out and isolated from each other. Interview informants perceived this isolation as an impediment to sociability, and in turn, cooperation.

Owners described the deterioration of rural, natural resource-based economies as a barrier to cooperation. Although formal cooperatives have never been pervasive among NIPF owners in the West (Kittredge 2005), agricultural cooperatives have served the practical need of connecting isolated rural residents with external markets, political processes, and each other (Hobbs 1995). With the decline in timber, cattle and other commodity markets, the basis for interaction and reciprocity among rural landowners in eastern Oregon has become scarce. Moreover, as communities of place are being incorporated into wider market economies and supplanted by social networks that are not geographically based, people may be less inclined to rely on local residents and resources (Brown 1993). Some theories suggest that less bounded contexts discourage cooperation because individuals are less likely to anticipate reciprocity due to remote relationships (Cohen and others 2001).

The demographic change associated with this shift in the rural economy may be further alienating landowners. In some areas of Oregon’s east side, affluent, retired, and otherwise mobile urbanites have migrated to rural areas for their amenities, bringing new values and expectations for land that can come into conflict with those of locals (Egan and Luloff 2000). The more recent rise of property individualism (Singer 2000) and increasing focus on privacy among forest owners (Butler 2008) also run counter to cooperation. Landowners’ fears of losing autonomy or control of their properties have been well-documented (Ellefson 2000; Fischer and Bliss 2009). For some, sharing information or inviting people over to discuss forest conditions and management may contradict values for privacy. Even poking one’s head over a fence to comment on conditions about which one is concerned is an invasion of privacy, as evidenced in the adage “good fences make good neighbors.”

Without membership to a common community or social group, landowners lack the structural and cultural basis for developing norms of reciprocity. Without interaction, they lack capacity to communicate and social mechanisms for developing trust among individuals. These are key conditions for cooperation (Ostrom 1990; Yaffee 1998; Tyler and Degoey 1995). Lack of group identity not only reduces interaction among landowners, it may also cause the lack of shared cognition about wildfire risk that owners said make cooperation difficult.

### Legitimacy

Although we found that some cooperation among private forest owners and public agencies occurs, many owners we interviewed reported cumbersome bureaucratic processes, corrosive expert-lay person relationships, and a lack of trustworthy leadership in natural resource management efforts that involved public agencies, which discouraged them from cooperating. Other research has shown that NIPF owners’ concerns about allowing government representatives onto their property, and agreeing to accept agency assistance lead to struggles over private property rights and undermine cooperation (Fischer and Bliss 2009). These concerns arise from owners’ perceptions of the legitimacy of public agencies. If people view an institution as legitimate they develop a voluntary sense of obligation to obey decisions, follow rules, or abide by social arrangements rather than doing so out of fear of punishment or anticipation of reward (Tyler 2006). This feeling of obligation is essential for successful cooperation.

### Risks and Benefits in Social Exchange

Survey results indicated that cooperation in fire hazard reduction does not occur frequently among private owners, yet many of the owners we interviewed said they communicated and cooperated frequently with other owners to address other land management problems. This discrepancy provides evidence that cooperation on fuel reduction depends on the benefits of social exchange outweighing the costs. In reciprocal social exchanges, the risk of betrayal is high (Cropanzano and Mitchell 2005). The potential for misunderstanding or failure to meet expectations of reciprocity may explain why owners infrequently cooperated with each other, despite a future willingness to do so. Perhaps some forms of cooperation—such as moving cattle and equipment onto each other’s property, and suppressing fires that have ignited—have benefits that outweigh the risk and inconvenience of working together. In contrast, the benefits of cooperation in fuel reduction are less certain given the mismatch in the nature of the transaction. Furthermore, it may be easier for parties to agree about things like relocating cattle and suppressing wildfires (shared cognition), than about fire risk mitigation, which invokes judgments about how well people manage land and protect others from risk.

Although there are substantial risks associated with cooperation between NIPF owners and public agencies, these social exchanges are generally negotiated, with both parties agreeing to a set of rules regarding commitments and expectations. In addition, substantial incentives exist for private–public cooperation, for example, when federal agencies offer cost-share monies, administrative and technical support, and other opportunities. In contrast, few policies or programs encourage or reward cooperation among private owners. These factors may help explain why owners have cooperated more frequently with public agencies than with each other.

## Models for Cooperative Wildfire Risk Management

The fact that so many owners expressed a willingness to cooperate with other private and public owners in the future despite limited past experience and recognized constraints; and the fact that about half already belong to organized, natural resource-related groups, suggests the potential for cooperation in landscape-scale forest management. Perceived fire risk alone may not compel owners to cooperate, but other policy and institutional incentives might. Interview informants identified a range of potential formal and informal models for cooperation. The tension between the informal and formal models lies in the need for flexible, low-pressure arrangements as well as coordination and efficiency. Some owners were willing to cooperate on an ad hoc basis; others wanted cooperation to be formally organized so that it would be efficient and ensure a benefit. Owners suggested that among neighbors, informal models may be preferable because they are less likely to make people feel rigid and defensive. Although owners described “over the fence”, “wheel and spoke” and “local group” models, we found only a few examples of these models operating in the context of fuel reduction in our study.

Despite owners’ beliefs about the importance of cooperation, and in light of the apparent lack of cooperation among owners, a less risky approach to cooperation among neighboring landowners may be one in which fuel reduction occurs through formal institutions (Cropanzano and Mitchell 2005). For example, the high cost of removing woody biomass and small-diameter logs, and lack of financial assistance and markets for this material are commonly identified barriers to fuel reduction (Fischer 2011). Formal institutional arrangements that enable owners to jointly apply for cost-share funds, coordinate treatments, and collectively offer biomass to the market could increase the economy of scale of management activities (Goldman and others 2007). Owners also identify liability and free ridership as drawbacks of cooperative fuel reduction. Formal institutions that coordinate management actions and pool risk can offer protection against liability and other risks associated with working with others (Amacher and others 2003).

Evidence exists for the emergence of new institutions that may offer an alternative path to addressing fire risk in Oregon and elsewhere in the western United States. Local collaborative institutions can provide an organized process for increasing the efficiency and focus of collaborative efforts without the binding terms that seem to put NIPF owners on edge. For example, Community Wildfire Protection Plans (CWPPs), established under the Healthy Forest Restoration Act, are tools for involving communities in fire risk mitigation on federal and nonfederal lands. They are funded by states but developed and implemented locally. While CWPP planning and implementation efforts don’t always reach beyond wildland-urban interface (WUI) boundaries and engage rural forestland owners, they have brought together many stakeholders and built relationships among community members around the issue of fire risk (Jakes and others 2007).

In California, Fire Safe Councils (that implement CWPPs in that state) have been recognized for their ability to promote innovative fire mitigation activities and build social capital in WUI communities (Everett and Fuller 2011). In Oregon, the nonprofit group Sustainable Northwest is working with landowner associations to expand processing facilities and develop merchandising yards for small-diameter wood, and to promote woody biomass heating systems (Sustainable Northwest 2011). Collaborative institutions such as these create the opportunity for frequent and sustained interaction among landowners having diverse motivations and values, a necessary foundation for building shared cognition, norms of reciprocity, and in cases where public agencies are involved, legitimacy (Bodin and others 2006).

Other cooperative models that could involve NIPF owners include The Nature Conservancy’s Fire Learning Network, and the U.S. Forest Service’s Collaborative Forest Landscape Restoration Program (CFLRP). Fire Learning Networks are regional groups that bring together public agencies, tribes, and municipal governments (though not specifically private forest owners) to plan and coordinate fuel reduction and forest restoration activities across ownerships. The CFLRP provides funding to local collaborative groups to plan science-based, economically viable fuel reduction and ecological restoration activities on select national forest lands. Although focused on federal lands, these efforts may be attractive to private forest owners if they help reduce the costs of, or create returns on, treatments on other ownerships, or decrease the legal risks associated with treatments through Memorandums of Understanding and formal partnerships. Future research could explore such models and the opportunities they offer for collective action for landscape-scale ecosystem management across ownership boundaries.

## Conclusion

In articulating his vision for America’s forests, U.S. Secretary of Agriculture Tom Vilsack has emphasized an “all lands approach” to forest restoration that calls for collaboration in undertaking landscape-scale restoration activities. Cooperation across ownership boundaries in fire prone, mixed-ownership forest landscapes is desirable yet challenging. Most of the NIPF landowners interviewed and surveyed for this study were concerned about fire risk on their lands and hazardous fuel conditions on the properties around them (and on public lands in particular), and treated fuel on their properties to reduce this risk. Although NIPF owners indicated a substantial willingness to cooperate with others on fuel reduction activities in the future, their past behavior demonstrated limited cooperation. Perceived risk of fire occurring on one’s property, and from nearby public forestlands were predictors of cooperation in fuel reduction with public land management agencies. Risk perception was not associated with cooperation among private landowners. The availability of funding and technical assistance from public agencies to help support fuel reduction on private lands, the greater social barriers to private–private cooperation than to private–public cooperation, and perceptions of more hazardous forest conditions on public lands relative to private lands may explain this difference.

Interview data suggest that social values and norms about property ownership work against cooperation, especially among NIPF owners, even when they perceive a risk of fire to their properties. Nevertheless, cooperation does occur among private owners in arenas other than fuel reduction—and it may occur indirectly through third parties, such as private contractors. Furthermore, owners say they are willing to cooperate with one another in the future. Thus, given the benefits of cooperation for landscape-scale natural resource management, new institutional models of cooperation to manage landscape-scale fire risk may hold promise.

From a policy standpoint, building a common understanding of fire risk among landowners, including fire risk on lands beyond their own property boundaries, may increase the likelihood that landowners will cooperate with others to reduce hazardous fuel. Promoting this awareness among landowners who reside on their properties may be particularly effective given the positive association between residing on one’s parcel and cooperation. Nevertheless, in the absence of policies and institutions that improve the balance between the costs of cooperation and the benefits of protecting one’s property from fire, cooperative landscape-scale management of natural hazards across ownership boundaries will be limited.
